# The Pleiotropic Effect of ANRIL in Glaucoma and Cardiovascular Disease

**DOI:** 10.3390/biomedicines13071617

**Published:** 2025-07-01

**Authors:** Luke O’Brien, Daire J. Hurley, Michael O’Leary, Liam Bourke, Colm O’Brien

**Affiliations:** Department of Ophthalmology, Mater Misericordiae University Hospital, Eccles Street, D07 R2WY Dublin, Ireland

**Keywords:** GWAS, SNP, glaucoma, CVD, ANRIL

## Abstract

**Background/Objectives**: The INK4 locus at chromosome 9p21.3, encoding CDKN2A, CDKN2B and the long non-coding RNA CDKN2B-AS1 (ANRIL), has been implicated in multiple diseases, including glaucoma and cardiovascular disease. ANRIL plays a critical role in gene regulation, inflammation and cell proliferation, contributing to disease susceptibility through shared molecular mechanisms. This study aims to identify SNPs within the INK4 locus associated with both glaucoma and CVD using the Open Targets Genetics platform and assess their pleiotropic effects. **Methods**: We utilised the Open Targets Genetics platform to identify SNPs at the INK4 locus associated with glaucoma and CVD. For each SNP, we recorded its genomic location, statistical significance and associated phenotypes. We further analysed the SNPs using the Genome Aggregation Database (gnomAD) to confirm their genomic position. Phenotypic associations were assessed using PheWAS data. **Results**: We identified 20 GWAS SNPs significantly associated with both glaucoma and CVD. All SNPs were located within intronic regions of the long non-coding RNA ANRIL. Certain SNPs such as rs4977756, rs1333037 and rs1063192 have known pleiotropic effects, influencing retinal ganglion cell survival in glaucoma and vascular smooth muscle cell proliferation in CVD. These SNPs influence shared biological pathways, including inflammation, oxidative stress and epigenetic regulation, and may exert either protective or pathogenic effects. Certain SNPs such as rs7853090 and rs1434537531 remain underexplored, emphasising the need for further research. **Conclusions**: This study highlights the pleiotropic role of ANRIL in glaucoma and CVD, driven by shared genetic and molecular pathways. While SNPs within ANRIL provide valuable insights into disease mechanisms, these conditions remain complex, influenced by multiple genetic and environmental factors. Targeting ANRIL therapeutically poses challenges due to its non-coding nature, but emerging RNA-based therapies, including antisense oligonucleotides and small-molecule modulators, hold promise. Further research into underexplored SNPs and ANRIL’s regulatory mechanisms is essential for advancing therapeutic development and understanding these multifactorial diseases.

## 1. Introduction

Population-based studies have demonstrated higher levels of cardiovascular disease (CVD) in patients with glaucoma. A nationwide cohort study in Korea found that glaucoma suspects with CVD had a greater risk of developing primary open-angle glaucoma (POAG) compared to those without CVD [1]. A study analysing the 2019 California Medicare population revealed that individuals with glaucoma had a statistically significant increased risk of CVD compared to individuals without glaucoma [2]. This association is seen in other large cohort studies including the UK Biobank Study (2023) and the Arterial Stiffness and Glaucoma Study (2024) [3,4].

A genome-wide association study (GWAS) is a population-based study that involves analysing the genomes of large groups of individuals to identify genetic variations associated with specific traits or diseases [5]. GWAS compare the genomes of individuals with the trait or disease (cases) to those without it (controls), focusing on single-nucleotide polymorphisms (SNPs). An SNP is a common genetic variation that involves a change in a single nucleotide at a specific position in the DNA sequence [6]. For example, at a particular genomic location, some individuals might have an adenine (A) while others have a guanine (G).

By analysing the frequency of each SNP between two groups, it is possible to identify genetic loci associated with a given trait. These loci may influence biological pathways, regulate gene expression or alter protein function, thereby contributing to the observed phenotype. Depending on their impact, they can exert either pathogenic or protective effects [5,6,7].

GWAS have advanced the understanding of genetic factors contributing to complex diseases such as glaucoma and CVD [5,8]. The INK4 locus at chromosome 9p21.3 has emerged as a critical genetic region associated with both glaucoma and CVD [9,10]. This locus includes key genes such as CDKN2A (Cyclin-Dependent Kinase Inhibitor 2A), CDKN2B (Cyclin-Dependent Kinase Inhibitor 2B) and the non-coding RNA CDKN2B-AS1, known as ANRIL (Antisense Noncoding RNA in the INK4 Locus). CDKN2A and CDKN2B both encode cyclin-dependent kinase inhibitors that regulate the cell cycle, preventing the transition from the G1 to S phase [11].

ANRIL regulates the expression of CDKN2A and CDKN2B and influences inflammation and vascular remodelling [12,13,14]. In CVD, dysregulated ANRIL contributes to plaque formation, instability and vascular inflammation [15]. In glaucoma, ANRIL plays a critical role in regulating neuronal survival and inflammatory pathways, contributing to optic nerve damage and disease progression [16].

**Figure 1:**
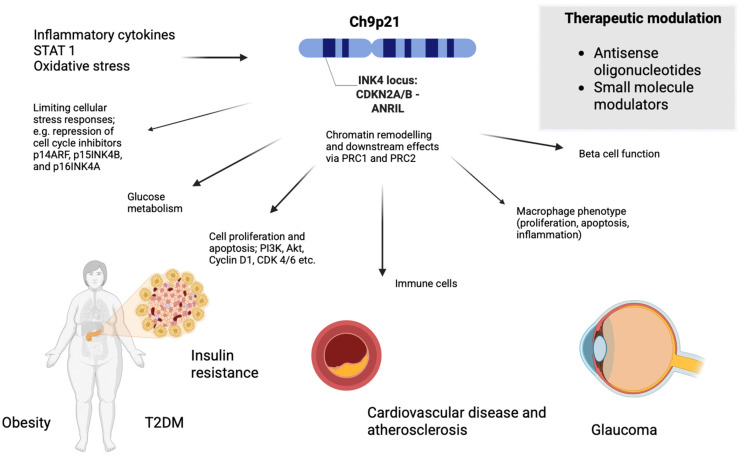
Pleiotropic effects of the INK4 locus across age-related chronic disease.

As illustrated in Figure 1, the INK4 locus plays a central and multifaceted role in disease pathogenesis by regulating cellular stress responses, metabolic function, immune activity and specific aspects of macrophage behaviour—including proliferation, apoptosis and inflammatory signalling [17]. This links the INK4 locus not only to CVD and glaucoma, but also to metabolic disorders including obesity and type 2 diabetes mellitus (T2DM) as well as to various cancers, further emphasising its central role in chronic disease pathogenesis [18,19].

**Figure 2:**
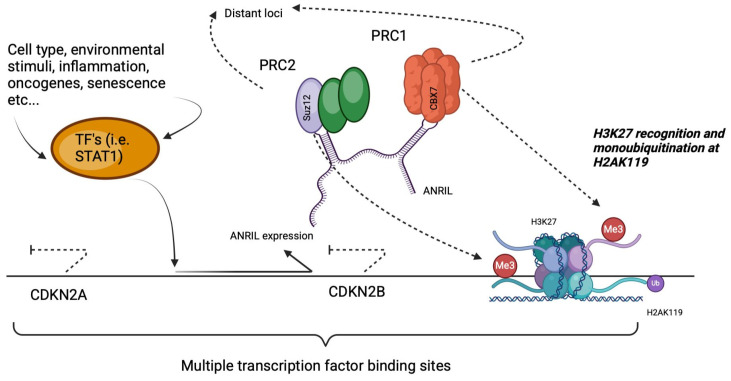
Regulation of the INK4 locus by ANRIL and Polycomb repressive complexes.

ANRIL expression itself is dynamically controlled by various stimuli such as inflammatory signals, environmental stress and transcription factors including STAT1 (Figure 2) [19]. Mechanistically, ANRIL acts as a scaffold for Polycomb repressive complexes PRC1 and PRC2, which mediate chromatin remodelling through histone modifications, notably H3K27 trimethylation and H2AK119 monoubiquitination [19]. These epigenetic changes suppress the transcription of nearby tumour-suppressor genes CDKN2A and CDKN2B, thereby linking upstream signals to downstream cell cycle and stress response pathways relevant to both glaucoma and CVD [20,21].

‘Open Targets Genetics’ (https://genetics.opentargets.org accessed on 12 January 2025) is an open access platform designed by Ghoussaini et al. that aggregates human GWAS and functional genomics data to make connections between GWAS-associated loci, gene variants and likely causal genes [22]. It was designed to assist with the discovery of suitable gene targets for drug development. The platform enables systematic identification and prioritisation of likely causal variants and genes across all published trait-associated loci. Open Targets Genetics uses a machine learning method to identify the most likely causal genes using a model known as the Locus 2 Gene (L2G) model as described by Ghoussaini et al. [22].

The aim of this study was to utilise the publicly available Open Targets platform to identify GWAS SNPs at the INK4 locus associated with glaucoma and to check whether these SNPs were shown to also be associated with CVD. Glaucoma comprises a group of optic neuropathies—including primary open-angle glaucoma (POAG), angle-closure glaucoma and normal-tension glaucoma (NTG)—characterised by retinal ganglion cell loss and progressive optic nerve degeneration [23]. Similarly, CVD encompasses conditions such as coronary artery disease, stroke and peripheral vascular disease, unified by common vascular risk factors and pathophysiological mechanisms [24]. While these subtypes differ clinically, growing evidence indicates shared underlying molecular pathways. Therefore, we analysed genetic associations using broad disease categories of ‘glaucoma’ and ‘cardiovascular disease’ rather than investigating individual subtypes. This approach aligns with the Open Targets Genetics platform, which classifies diseases into broad groups, facilitating identification of shared genetic variants. Notably, most glaucoma GWAS data derive from studies of POAG and NTG, making these subtypes the primary contributors to associations within the broad ‘glaucoma’ category [25]. Similarly, the CVD category is predominantly informed by GWAS on major events such as myocardial infarction, coronary artery disease and ischemic stroke.

To our knowledge, this work represents a novel application of the Open Targets Genetics platform to systematically identify shared pleiotropic SNPs within ANRIL for glaucoma and CVD.

## 2. Materials and Methods

Using the Open Targets platform (https://genetics.opentargets.org), we performed separate searches for two of the protein-coding genes found at the INK4 locus (CDKN2A and CDKN2B) [22]. Searching for ‘CDKN2A’ on the Open Targets platform generates information including the gene location, a list of studies associated with CDKN2A from the locus-to-gene pipeline and a list of studies which have evidence of co-localisation of CDKN2A via quantitative trait locus (QTL) analysis. In the top right-hand corner of the webpage, there is a box which gives the option to ‘view CDKN2A in the Open Targets Platform’. Further information on the gene is displayed including a list of tissues that have been shown to have various levels of CDKN2A RNA and protein expression, gene ontology information, genetic constraint information, ProtVista data (functional, positional and structural protein information), data on molecular interactions, biological pathways in which it is involved and mouse phenotypes associated with CDKN2A. Towards the top left of the page, there is an option to select ‘associated diseases’. A list is displayed of all diseases shown to be associated with CDKN2A. Using the search box, we performed a search for ‘glaucoma’. By selecting the ‘OT Genetics’ option, a list is generated of GWAS variants which prioritise CDKN2A as the likely causal gene for glaucoma. Each gene variant is displayed along with the study source and publication. By clicking on each variant, you are brought to a page displaying information including the genomic location, nearest gene, a list of genes functionally impaired by the variant and a list of traits that are associated with this variant in the UK Biobank, FinnGen and/or GWAS Catalog. Only traits with *p*-value < 0.005 are displayed. A list is also generated of the GWA studies in which the variant is featured along with the phenotype/trait, *p*-value, odds ratio and the number of cases in the study. We recorded the details of the studies that were the most statistically significant for the phenotypes of glaucoma and CVD for each gene variant. We then used ‘The Genome Aggregation Database’ (https://gnomad.broadinstitute.org/about accessed on 24 January 2025) to search for each SNP and record further information including the genomic location of each SNP [26]. The Genome Aggregation Database is a publicly accessible resource maintained by the Broad Institute of MIT and Harvard, providing population-wide allele frequencies and annotations for genetic variants to support academic and clinical research. This process was then repeated for the other protein-coding gene CDKN2B. Due to Open Targets Genetics being designed to assist with drug discovery, it focuses on protein-coding genes. Since ANRIL is a long non-coding RNA (lncRNA), it does not generate any results on the platform, although all of the GWAS gene variants generated from the CDKN2A and CDKN2B searches are found within the ANRIL genomic region.

## 3. Results

By carrying out the search as described in the Section 2 first for CDKN2A, we generated a list of 38 GWAS gene variants which were statistically associated with the glaucoma phenotype. After removing duplicate results, there were 20 GWAS variants remaining (Supplementary Table S1).

All 20 of these GWAS gene variants were SNPs. We recorded the ‘RS number’ (Reference SNP number) for each SNP—this is the unique identifier assigned to each SNP in the Database of Single Nucleotide Polymorphisms (dbSNP) [27]. The dbSNP is a comprehensive, publicly accessible database maintained by the National Centre for Biotechnology Information (NCBI). For each GWAS gene variant/SNP, we recorded the study source (UK Biobank, FinnGen or GWAS Catalog), the GWAS publication, the *p*-value for expressing the glaucoma phenotype from the GWAS with the highest level of statistical significance, the odds ratio (if available), the locus-to-gene score and the sample size (Supplementary Tables S2 and S3).

We studied each of the 20 SNPs in detail using the Open Targets Platform and examined the other phenotypes/traits associated with each SNP as per PheWAS—all of the 20 GWAS variants also prioritise CDKN2A as the likely causal gene for CVD with high levels of statistical significance (Supplementary Table S3) [22]. Using ‘The Genome Aggregation Database’ (https://gnomad.broadinstitute.org/about), we then performed a search for each SNP [23]. Of significance, all of the 20 SNPs are located within the genomic coordinates of the CDKN2B-AS1 gene (ANRIL) and are situated within its intronic (non-coding) region. The same 38 GWAS gene variant results were generated when the search was repeated for CDKN2B, with the same 20 SNPs remaining following removal of duplicates.

## 4. Discussion

SNPs are the most common type of genetic variation among humans [5,6,7]. They are scattered throughout the genome and can be found in both coding regions (exons) and non-coding regions (introns, promoters and intergenic areas) [5,6,7]. They are identified in populations through large-scale studies such as GWAS, which compare the genomes of individuals with a specific trait or disease (cases) to those without it (controls) [5,6,7]. SNPs can play varying roles—some may be neutral, while others influence biological processes. Depending on their location and effect, SNPs can be associated with either increased susceptibility to diseases (pathogenic SNPs) or provide protection against conditions (protective SNPs) [5,6,7]. For example, an SNP in a regulatory region might alter gene expression, contributing to pathology, while another might enhance resilience to disease [5,6,7]. The identification of SNPs and their functional implications is crucial for understanding genetic contributions to health and disease and for developing targeted therapies [5,6,7].

In this study, using a publicly available platform to access GWAS data, we have identified 20 SNPs that have been shown to be associated with both glaucoma and CVD with strong statistical significance. All 20 SNPs are located within the CDKN2B-AS1 (ANRIL) gene. ANRIL is a long non-coding RNA gene at the 9p21.3 locus, which is well-known for its association with multiple diseases, including glaucoma and CVD [9,10]. SNPs within ANRIL such as rs4977756, rs1333037 and rs1063192 have been shown to influence both glaucoma and CVD through overlapping molecular pathways [28,29,30,31,32,33]. ANRIL plays a central regulatory role in gene expression, interacting with nearby genes CDKN2A and CDKN2B, which are critical in maintaining cellular homeostasis, vascular integrity and neuronal survival [9,11]. Variants in ANRIL impact these processes by altering its transcription, splicing and interaction with chromatin-modifying complexes, leading to dysregulation of cell cycle progression, inflammation and epigenetic modifications [14,20].

In glaucoma, ANRIL SNPs contribute to optic nerve degeneration by influencing RGC survival and neuroinflammatory pathways [13,16,34]. Studies have demonstrated that rs1063192 is significantly associated with primary open-angle glaucoma (POAG) and impacts vertical cup-to-disc ratio, a key glaucoma endophenotype [35,36]. It has been shown to be associated with a smaller vertical cup-to-disc ratio and thus have a protective effect against glaucoma [35,36]. Similarly, rs4977756 has been strongly linked to POAG and has been shown to influence both central corneal thickness and vertical cup-to-disc-ratio [28].

In CVD, ANRIL SNPs have been shown to play a role in co-ordinating tissue remodelling by modulating the expression of genes involved in cell proliferation, apoptosis, extra-cellular matrix remodelling and inflammation [37]. ANRIL SNPs including rs4977756 and rs7865618 have both been shown to be associated with coronary artery disease and myocardial infarction with statistical significance [29,38,39].

The shared biological mechanisms highlight the pleiotropic nature of the INK4 locus and ANRIL via their effects on cellular processes including inflammation, oxidative stress and vascular remodelling as demonstrated in Figure 1 [12,14].

It is important to note that while some SNPs, such as rs4977756 and rs1063192, have been extensively studied, others within ANRIL remain underexplored. For instance, SNPs such as rs7853090 and rs1434537531 have limited functional data available, highlighting the need for further research to uncover their potential roles in disease susceptibility and the regulation of ANRIL’s activity.

These findings emphasise ANRIL’s role as a central player in disease susceptibility and offer critical insights into its potential as a therapeutic target. Understanding the functional consequences of these SNPs could pave the way for innovative strategies to address both glaucoma and CVD through shared molecular pathways. Therapies aimed at modulating ANRIL expression, restoring vascular health or controlling inflammation could provide dual benefits in mitigating disease progression. Further research, particularly functional studies, is essential to elucidate the precise molecular mechanisms through which ANRIL exerts its effects and to translate these findings into clinical applications.

Targeting ANRIL therapeutically would likely be challenging due to its non-coding nature and complex regulatory functions [14,20]. As alluded to in Figure 1, potential strategies include the use of antisense oligonucleotides (ASOs) or RNA interference (RNAi) to suppress ANRIL transcription or splicing, and CRISPR/Cas technologies to modulate its activity or correct disease-associated variants [40]. Small-molecule modulators could disrupt ANRIL’s interactions with chromatin modifiers such as PRC1 and PRC2, while epigenetic drugs such as histone deacetylase (HDAC) inhibitors could restore balanced gene regulation at the INK4 locus [19,20,41].

While SNPs within ANRIL and the INK4 locus provide valuable insights into genetic susceptibility for diseases such as glaucoma and CVD, these conditions are highly complex and influenced by multiple factors. Environmental and lifestyle elements, such as diet, exercise, smoking and systemic health conditions such as diabetes and hypertension, play significant roles in disease risk and progression. In addition to these factors, emerging evidence highlights the critical role of circadian rhythms in modulating inflammation and oxidative stress—two key processes influenced by ANRIL [42,43]. Disruptions in circadian cycles, which regulate physiological and cellular functions in a time-dependent manner, have been linked to exacerbated inflammatory responses and increased oxidative damage. Notably, intraocular pressure (IOP), a major risk factor for glaucoma, exhibits a distinct circadian pattern, typically peaking in the early morning hours and declining throughout the day [44]. Similarly, blood pressure, an important factor in cardiovascular disease, follows a circadian rhythm with higher levels in the morning [45]. Given the involvement of light-sensitive retinal ganglion cells in glaucoma pathogenesis and the systemic nature of cardiovascular disease, the interplay between circadian regulation and ANRIL’s pleiotropic effects represents an important, yet underexplored, dimension. Integrating circadian biology into future research could provide valuable insights into the temporal regulation of ANRIL-mediated pathways, potentially revealing novel therapeutic windows and strategies for managing both glaucoma and CVD.

Glaucoma and CVD are complex polygenic diseases involving contributions from many genes beyond ANRIL, as well as gene–gene and gene–environment interactions. Epigenetic modifications further complicate the landscape, as they can modulate gene expression in response to environmental exposures [21]. Together, this highlights that while genetic variants such as those within ANRIL are important, they represent only part of the multifactorial nature of these conditions, emphasising the need for a holistic approach to understanding and managing these diseases.

While our analysis included GWAS data from multiple sources—including the GWAS Catalog (*n* = 13), FinnGen (*n* = 3), UK Biobank (*n* = 2) and combined datasets (*n* = 2)—we acknowledge that many of these studies predominantly involved individuals of European ancestry. Although the GWAS Catalog incorporates data from diverse populations, including East Asian, African, South Asian and Hispanic/Latino cohorts, the overall representation remains limited. As linkage disequilibrium (LD) patterns and SNP–disease associations can vary between populations, future studies incorporating more ancestrally diverse cohorts and performing ancestry-stratified analyses will be essential to validate and expand upon these findings.

## 5. Conclusions

In this study, we uniquely applied the Open Targets Genetics platform to systematically identify pleiotropic SNPs within ANRIL shared between glaucoma and cardiovascular disease. Our findings highlight the complex pleiotropic nature of ANRIL and its involvement in key cellular processes relevant to both conditions. These results offer novel insights into the shared genetic architecture of glaucoma and CVD and suggest potential avenues for future research and therapeutic development.

## Data Availability

Data are contained within the article and Supplementary Materials.

## Supplementary Materials

The following supporting information can be downloaded at https://www.mdpi.com/article/10.3390/biomedicines13071617/s1: Table S1: GWAS variants (SNPs) which prioritise CDKN2A and CDKN2B as the causal genes for glaucoma; Table S2: Details of each GWAS variant for the glaucoma phenotype; Table S3: GWAS variants’ association with CVD and glaucoma. References [46,47,48,49,50] are cited in Supplementary Materials.

## Abbreviations

The following abbreviations are used in this manuscript:

CVD	Cardiovascular disease
SNP	Single-nucleotide polymorphism
POAG	Primary open-angle glaucoma
GWAS	Genome-wide association study
CDKN2A	Cyclin-Dependent Kinase Inhibitor 2A
CDKN2B	Cyclin-Dependent Kinase Inhibitor 2B
RGC	Retinal ganglion cell
T2DM	Type 2 diabetes mellitus
PRC	Polycomb recessive complex
dbSNP	Database of Single Nucleotide Polymorphisms
ANRIL	Antisense Noncoding RNA in the INK4 Locus

## References

[1] Jung K.I., Kim Y.C., Shin H.J., Park C.K. (2025). Nationwide cohort study of primary open angle glaucoma risk and cardiovascular factors among in Korean glaucoma suspects. Sci. Rep..

[2] Emami S., Yui F., Coleman A.L. (2022). The Association Between Glaucoma and Cardiovascular Disease in the Elderly California Medicare Population. Investig. Ophthalmol. Vis. Sci..

[3] Choi J.A., Lee S.N., Jung S.H., Won H.H., Yun J.S. (2023). Association of glaucoma and lifestyle with incident cardiovascular disease: A longitudinal prospective study from UK Biobank. Sci. Rep..

[4] Beros A.L., Sluyter J.D., Hughes A.D., Hametner B., Wassertheurer S., Scragg R.K.R. (2024). Arterial Stiffness and Incident Glaucoma: A Large Population-Based Cohort Study. Am. J. Ophthalmol..

[5] Visscher P.M., Wray N.R., Zhang Q., Sklar P., McCarthy M.I., Brown M.A., Yang J. (2017). 10 Years of GWAS Discovery: Biology, Function, and Translation. Am. J. Hum. Genet..

[6] Brookes A.J. (1999). The essence of SNPs. Gene.

[7] Tam V., Patel N., Turcotte M., Bossé Y., Paré G., Meyre D. (2019). Benefits and limitations of genome-wide association studies. Nat. Rev. Genet..

[8] Hysi P.G., Cheng C.Y., Springelkamp H., Macgregor S., Bailey J.N., Wojciechowski R., Vitart V., Nag A., Hewitt A.W., Höhn R. (2014). Genome-wide analysis of multi-ancestry cohorts identifies new loci influencing intraocular pressure and susceptibility to glaucoma. Nat. Genet..

[9] Cunnington M.S., Koref M.S., Mayosi B.M., Burn J., Keavney B. (2010). Chromosome 9p21 SNPs associated with multiple disease phenotypes correlate with ANRIL expression. PLoS Genet..

[10] Burdon K.P., Macgregor S., Hewitt A.W., Sharma S., Chidlow G., Mills R.A., Danoy P., Casson R., Viswanathan A.C., Liu J.Z. (2011). Genome-wide association study identifies susceptibility loci for open angle glaucoma at TMCO1 and CDKN2B-AS1. Nat. Genet..

[11] Sherr C.J., Roberts J.M. (1999). CDK inhibitors: Positive and negative regulators of G 1-phase. Genes Dev..

[12] Kuo C.L., Murphy A.J., Sayers S., Li R., Yvan-Charvet L., Davis J.Z., Krishnamurthy J., Liu Y., Puig O., Sharpless N.E. (2011). Cdkn2a is an atherosclerosis modifier locus that regulates monocyte/macrophage proliferation. Arterioscler. Thromb. Vasc. Biol..

[13] Pasquale L.R., Loomis S.J., Kang J.H., Yaspan B.L., Abdrabou W., Budenz D.L., Chen T.C., DelBono E., Friedman D.S., Gaasterland D. (2013). CDKN2B-AS1 genotype-glaucoma feature correlations in primary open-angle glaucoma patients from the United States. Am. J. Ophthalmol..

[14] Congrains A., Kamide K., Ohishi M., Rakugi H. (2013). ANRIL: Molecular mechanisms and implications in human health. Int. J. Mol. Sci..

[15] Holdt L.M., Beutner F., Scholz M., Gielen S., Gäbel G., Bergert H., Schuler G., Thiery J., Teupser D. (2010). ANRIL expression is associated with atherosclerosis risk at chromosome 9p21. Arterioscler. Thromb. Vasc. Biol..

[16] Gauthier A.C., Liu J. (2017). Epigenetics and Signaling Pathways in Glaucoma. BioMed Res. Int..

[17] Pellarin I., Dall’Acqua A., Favero A., Segatto I., Rossi V., Crestan N., Karimbayli J., Belletti B., Baldassarre G. (2025). Cyclin-dependent protein kinases and cell cycle regulation in biology and disease. Signal Transduct. Target. Therapy.

[18] Grarup N., Sandholt C.H., Hansen T., Pedersen O. (2014). Genetic susceptibility to type 2 diabetes and obesity: From genome-wide association studies to rare variants and beyond. Diabetologia.

[19] Aguilo F., Di Cecilia S., Walsh M.J. (2016). Long Non-coding RNA ANRIL and Polycomb in Human Cancers and Cardiovascular Disease. Curr. Top. Microbiol. Immunol..

[20] Kotake Y., Nakagawa T., Kitagawa K., Suzuki S., Liu N., Kitagawa M., Xiong Y. (2011). Long non-coding RNA ANRIL is required for the PRC2 recruitment to and silencing of p15 INK4B tumor suppressor gene. Oncogene.

[21] Bure I.V., Nemtsova M.V., Kuznetsova E.B. (2022). Histone Modifications and Non-Coding RNAs: Mutual Epigenetic Regulation and Role in Pathogenesis. Int. J. Mol. Sci..

[22] Ghoussaini M., Mountjoy E., Carmona M., Peat G., Schmidt E.M., Hercules A., Fumis L., Miranda A., Carvalho-Silva D., Buniello A. (2021). Open Targets Genetics: Systematic identification of trait-associated genes using large-scale genetics and functional genomics. Nucleic Acids Res..

[23] Weinreb R.N., Aung T., Medeiros F.A. (2014). The Pathophysiology and Treatment of Glaucoma. JAMA.

[24] Olvera Lopez E., Ballard B.D., Jan A. (2025). Cardiovascular Disease.

[25] Gharahkhani P., Jorgenson E., Hysi P., Khawaja A.P., Pendergrass S., Han X., Ong J.S., Hewitt A.W., Segrè A.V., Rouhana J.M. (2021). Genome-wide meta-analysis identifies 127 open-angle glaucoma loci with consistent effect across ancestries. Nat. Commun..

[26] Karczewski K.J., Francioli L.C., Tiao G., Cummings B.B., Alföldi J., Wang Q., Collins R.L., Laricchia K.M., Ganna A., Birnbaum D.P. (2020). The mutational constraint spectrum quantified from variation in 141,456 humans. Nature.

[27] Sherry S.T., Ward M.H., Kholodov M., Baker J., Phan L., Smigielski E.M., Sirotkin K. (2001). dbSNP: The NCBI database of genetic variation. Nucleic Acids Res..

[28] Rathi S., Danford I., Gudiseva H.V., Verkuil L., Pistilli M., Vishwakarma S., Kaur I., Dave T.V., O’Brien J.M., Chavali V.R. (2020). Molecular genetics and functional analysis implicate cdkn2bas1-cdkn2b involvement in poag pathogenesis. Cells.

[29] Xu B., Xu Z., Chen Y., Lu N., Shu Z., Tan X. (2021). Genetic and epigenetic associations of ANRIL with coronary artery disease and risk factors. BMC Med. Genom..

[30] Zukerman R., Harris A., Vercellin A.V., Siesky B., Pasquale L.R., Ciulla T.A. (2021). Molecular genetics of glaucoma: Subtype and ethnicity considerations. Genes.

[31] Vargas J.D., Manichaikul A., Wang X.Q., Rich S.S., Rotter J.I., Post W.S., Polak J.F., Budoff M.J., Bluemke D.A. (2016). Detailed analysis of association between common single nucleotide polymorphisms and subclinical atherosclerosis: The Multi-ethnic Study of Atherosclerosis. Data Brief..

[32] Hu Z., He C. (2017). CDKN2B gene rs1063192 polymorphism decreases the risk of glaucoma. Oncotarget.

[33] Liu R., Song L., Jiang L., Tang X., Xu L., Gao Z., Zhao X., Xu J., Gao R., Yuan J. (2020). Susceptible gene polymorphism in patients with three-vessel coronary artery disease. BMC Cardiovasc. Disord..

[34] Danford I.D., Verkuil L.D., Choi D.J., Collins D.W., Gudiseva H.V., Uyhazi K.E., Lau M.K., Kanu L.N., Grant G.R., Chavali V.R. (2017). Characterizing the “POAGome”: A bioinformatics-driven approach to primary open-angle glaucoma. Prog. Retin. Eye Res..

[35] Fan B.J., Wang D.Y., Pasquale L.R., Haines J.L., Wiggs J.L. (2011). Genetic variants associated with optic nerve vertical cup-to-disc ratio are risk factors for primary open angle glaucoma in a US Caucasian population. Investig. Ophthalmol. Vis. Sci..

[36] Ramdas W.D., van Koolwijk L.M., Lemij H.G., Pasutto F., Cree A.J., Thorleifsson G., Janssen S.F., Jacoline T.B., Amin N., Rivadeneira F. (2011). Common genetic variants associated with open-angle glaucoma. Hum. Mol. Genet..

[37] Congrains A., Kamide K., Katsuya T., Yasuda O., Oguro R., Yamamoto K., Ohishi M., Rakugi H. (2012). CVD-associated non-coding, R.N.A.; ANRIL; modulates expression of atherogenic pathways in VSMC. Biochem. Biophys. Res. Commun..

[38] AlRasheed M.M., Hefnawy M.M., Elsherif N.N., Alhawassi T.M., Abanmy N.O., Al Rasheed N.M., Alqahtani F.Y., Aleanizy F.S., Muiya P., Al-Boudari O.M. (2018). The role of CDKN2B in cardiovascular risk in ethnic Saudi Arabs: A validation study. Gene..

[39] Manjula G., Pranavchand R., Kumuda I., Reddy B.S., Reddy B.M. (2020). The SNP rs7865618 of 9p21.3 locus emerges as the most promising marker of coronary artery disease in the southern Indian population. Sci. Rep..

[40] Bennett C.F. (2025). Therapeutic Antisense Oligonucleotides Are Coming of Age. Annu. Rev. Med..

[41] Falkenberg K.J., Johnstone R.W. (2014). Histone deacetylases and their inhibitors in cancer, neurological diseases and immune disorders. Nat. Rev. Drug Discov..

[42] Wilking M., Ndiaye M., Mukhtar H., Ahmad N. (2013). Circadian Rhythm Connections to Oxidative Stress: Implications for Human Health. Antioxid. Redox Signal..

[43] Maiese K. (2018). Novel Treatment Strategies for the Nervous System: Circadian Clock Genes, Non-coding RNAs, and Forkhead Transcription Factors. Curr. Neurovasc Res..

[44] Konstas A.G., Kahook M.Y., Araie M., Katsanos A., Quaranta L., Rossetti L., Holló G., Detorakis E.T., Oddone F., Mikropoulos D.G. (2018). Diurnal and 24-h Intraocular Pressures in Glaucoma: Monitoring Strategies and Impact on Prognosis and Treatment. Adv. Ther..

[45] Zhang J., Sun R., Jiang T., Yang G., Chen L. (2021). Circadian Blood Pressure Rhythm in Cardiovascular and Renal Health and Disease. Biomolecules..

[46] Craig J.E., Han X., Qassim A., Hassall M., Cooke Bailey J.N., Kinzy T.G., Khawaja A.P., An J., Marshall H., Gharahkhani P. (2020). Multitrait analysis of glaucoma identifies new risk loci and enables polygenic prediction of disease susceptibility and progression. Nat. Genet..

[47] Neale B. (2018). UK Biobank GWAS Results. https://www.nealelab.is/uk-biobank.

[48] Dönertaş H.M., Fabian D.K., Fuentealba M., Partridge L., Thornton J.M. (2021). Common genetic associations between age-related diseases. Nat. Aging.

[49] van der Harst P., Verweij N. (2018). Identification of 64 Novel Genetic Loci Provides an Expanded View on the Genetic Architecture of Coronary Artery Disease. Circ. Res..

[50] Hartiala J.A., Han Y., Jia Q., Hilser J.R., Huang P., Gukasyan J., Schwartzman W.S., Cai Z., Biswas S., Tregouet D.A. (2021). Genome-wide analysis identifies novel susceptibility loci for myocardial infarction. Eur. Heart J..

