# Supplementary material for: The Pleiotropic Effect of ANRIL in Glaucoma and Cardiovascular Disease
Source: Biomedicines. 2025 Jul 1;13(7):1617. doi: 10.3390/biomedicines13071617 (PMC12292808; doi:10.3390/biomedicines13071617)
Supplement: Supplementary file 1 [file biomedicines-13-01617-s001.zip › biomedicines-3691988-supplementary.pdf]

Table S1. GWAS variants (SNPs) which prioritise CDKN2A and CDKN2B as the causal gene for glaucoma.

GWAS SNP	RS Number
9_22051671_G_C	rs944801
9_22040766_C_T	rs1333037
9_22056360_A_G	rs7866783
9_22033367_C_T	rs2157719
9_22052735_T_C	rs6475604
9_22032153_T_G	rs634537
9_22050899_A_G	rs944800
9_22056296_T_C	rs7853090
9_22047946_T_C	rs10120806
9_22049657_G_A	rs10811645
9_22043927_A_G	rs1412829
9_22053957_TA_T	rs1434537531
9_22068653_G_A	rs4977756
9_22003368_G_A	rs1063192
9_22049483_C_CTT	rs34713115
9_22024024_A_T	rs504318
9_22032120_A_G	rs10115049
9_22028802_A_G	rs7049105
9_22031006_G_A	rs7865618
9_22019130_A_G	rs523096

Table S2. Details of each GWAS variant for the glaucoma phenotype.

Locus/Variant ID	RS Number	Study Source	Glaucoma Phenotype (p-value and study)	oddsRatioCi95	l2GScore	sampleSize
9_22051671_G_C	rs944801	GWAS Catalog, UK Biobank	p < 2.0e-38 - Craig JE (2020)		0.8714836836	137086
9_22040766_C_T	rs1333037	GWAS Catalog	p < 3.0e-36 - Craig JE (2020)	1.67	0.8631402254	1870

9_22056360_A_G	rs7866783	GWAS Catalog	p < 3.4e-37 - Craig JE (2020)		0.8597713113	5090
9_22033367_C_T	rs2157719	GWAS Catalog	p < 2.6e-36 - Craig JE (2020)		0.8580571413	6633
9_22052735_T_C	rs6475604	GWAS Catalog, UK Biobank	p < 4.6e-38 - Craig JE (2020)		0.8523216844	402223
9_22032153_T_G	rs634537	GWAS Catalog	p < 4.0e-36 - Craig JE (2020)		0.8423284888	177351
9_22050899_A_G	rs944800	GWAS Catalog	p < 4.1e-24 - Craig JE (2020)	1.33	0.8290446401	22795
9_22056296_T_C	rs7853090	GWAS Catalog	p < 1.3e-35 - Craig JE (2020)	1.3153464096	0.8249402642	53141
9_22047946_T_C	rs10120806	GWAS Catalog	p < 9.7e-34 - Craig JE (2020)		0.8233862519	662330
9_22049657_G_A	rs10811645	GWAS Catalog	p < 2.1e-34 - Craig JE (2020)	1.1627907	0.8216416836	63412
9_22043927_A_G	rs1412829	GWAS Catalog	p < 3.0e-35 - Craig JE (2020)	0.717128617	0.8202751875	46523
9_22053957_TA_T	rs1434537531	UK Biobank	p < 3.2e-18 - UKB Neale v2 (2018)	1.2096690775	0.798417449	361141
9_22068653_G_A	rs4977756	GWAS Catalog	p < 3.7e-29 - Craig JE (2020)	1.39	0.7841208577	4446
9_22003368_G_A	rs1063192	GWAS Catalog	p < 2.8e-35 - Craig JE (2020)	1.33	0.7711452842	7993
9_22049483_C_CTT	rs34713115	UK Biobank	p < 2.2e-20 - Donertas HM (2021)	1.199057921	0.7519530654	117890
9_22024024_A_T	rs504318	FinnGen	p < 2.2e-25 - Craig JE (2020)	0.8988222354	0.6451846361	260405

9_22032120_A_G	rs10115049	FinnGen	p < 2.8e-32 - Craig JE (2020)	1.5352296823	0.6145458221	250980
9_22028802_A_G	rs7049105	FinnGen	p < 8.3e-33 - Craig JE (2020)	1.1898532736	0.6031907201	255283
9_22031006_G_A	rs7865618	GWAS Catalog	p < 1.4e-36 - Craig JE (2020)	1.78	0.5995910764	1519
9_22019130_A_G	rs523096	GWAS Catalog	p < 1.2e-30 - Craig JE (2020)	0.4694835681	0.5856201649	843

Table S3. GWAS variants association with CVD & glaucoma.

Locus/Variant ID	RS Number	CVD Phenotype (p-value and study)	Glaucoma Phenotype (p-value and study)
9_22051671_G_C	rs944801	p < 3.7e-68 - van der Harst P (2017)	p < 2.0e-38 - Craig JE (2020)
9_22040766_C_T	rs1333037	p < 3.4e-87 - van der Harst P (2017)	p < 3.0e-36 - Craig JE (2020)
9_22056360_A_G	rs7866783	p < 1.7e-71 - Van der Harst P (2017)	p < 3.4e-37 - Craig JE (2020)
9_22033367_C_T	rs2157719	p < 5.1e-84 - Van der Harst P (2017)	p < 2.6e-36 - Craig JE (2020)
9_22052735_T_C	rs6475604	p < 2.9e-71 - Van der Harst P (2017)	p < 4.6e-38 - Craig JE (2020)
9_22032153_T_G	rs634537	p < 4.7e-87 - Van der Harst P (2017)	p < 4.0e-36 - Craig JE (2020)
9_22050899_A_G	rs944800	p < 6.5e-72 - Van der Harst P (2017)	p < 4.1e-24 - Craig JE (2020)
9_22056296_T_C	rs7853090	p < 3.5e-71 - Van der Harst P (2017)	p < 1.3e-35 - Craig JE (2020)
9_22047946_T_C	rs10120806	p < 1.6e-76 - Van der Harst P (2017)	p < 9.7e-34 - Craig JE (2020)
9_22049657_G_A	rs10811645	p < 1.7e-75 - Van der Harst P (2017)	p < 2.1e-34 - Craig JE (2020)
9_22043927_A_G	rs1412829	p < 3.0e-87 - Van der Harst P (2017)	p < 3.0e-35 - Craig JE (2020)
9_22053957_TA_T	rs1434537531	p < 8.0e-22 - UKB Neale v2 (2018)	p < 3.2e-18 - UKB Neale v2 (2018)
9_22068653_G_A	rs4977756	p < 2.4e-101 - Van der Harst P (2017)	p < 3.7e-29 - Craig JE (2020)

9_22003368_G_A	rs1063192	p < 1.8e-64 - Vander Harst (2017)	p < 2.8e-35 - Craig JE (2020)
9_22049483_C_CTT	rs34713115	p < 8.6e-35 - Hartiala JA (2021)	p < 2.2e-20 - Donertas HM (2021)
9_22024024_A_T	rs504318	p < 1.1e-63 - Van der Harst P (2017)	p < 2.2e-25 - Craig JE (2020)
9_22032120_A_G	rs10115049	p < 3.4e-104 - Van der Harst P (2017)	p < 2.8e-32 - Craig JE (2020)
9_22028802_A_G	rs7049105	p < 5.6e-101 - Van der Harst (2017)	p < 8.3e-33 - Craig JE (2020)
9_22031006_G_A	rs7865618	p < 1.1e-68 - Van der Harst (2017)	p < 1.4e-36 - Craig JE (2020)
9_22019130_A_G	rs523096	p < 9.1e-55 - Van der Harst (2017)	p < 1.2e-30 - Craig JE (2020)
